# Supplementary material for: Identification of photoperiod-induced specific miRNAs in the adrenal glands of Sunite sheep (Ovis aries)
Source: Front Vet Sci. 2022 Jul 22;9:888207. doi: 10.3389/fvets.2022.888207 (PMC9354845; doi:10.3389/fvets.2022.888207)
Supplement: Supplementary file 1 [file Data_Sheet_1.ZIP › Supplementary file/miRDeep2 file.zip!/miRDeep2 file/Know_Structure/SPLP42A3/oar-mir-181a-2.pdf]

miRBase precursor	: oar-mir-181a-2
Total read count	: 15746
oar-miR-181a read count	: 15626
remaining reads	: 120

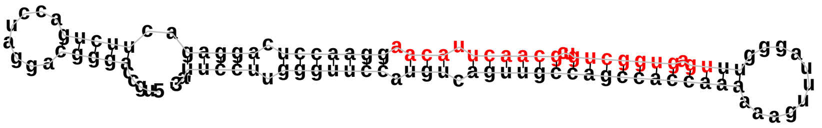

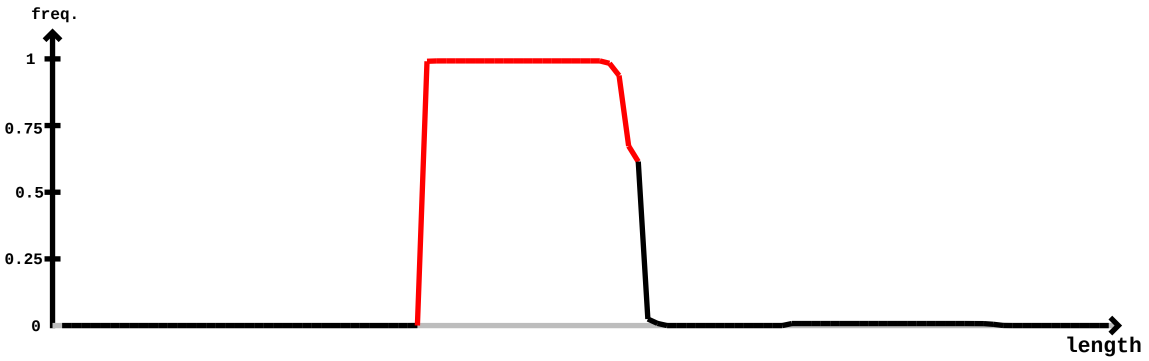


```
                                    oar-miR-181a
5'- ugccagggcaggauccagucuucagaggacuccaaggaacauucaacgcugucggugaguuugggauuugaaaaaaccaccgaccguugacuguaccuuggguuccuua   -3'    exp
    ....(((((........)))))..(((((.(((((((.(((.((((((..(((((((.((((...........))))))))))))))))).))).))))))).))))).   reads      mm         sample
    ....................................gaacauucaacgcugucgguga...................................................   3          0          seq
    .....................................aacauucaacgcugucggu.....................................................   140        0          seq
    .....................................aacauucaacgcugucggug....................................................   721        0          seq
    .....................................aacauucaacgcugucgguga...................................................   4156       0          seq
    .....................................aacauucaacgcugucggugag..................................................   918        0          seq
    .....................................aacauucaacgcugucggugagu.................................................   9280       0          seq
    .....................................aacauucaacgcugucggugaguu................................................   260        0          seq
    .....................................aacauucaacgcugucggugaguuu...............................................   127        0          seq
    ......................................acauucaacgcugucggu.....................................................   1          0          seq
    ......................................acauucaacgcugucggug....................................................   1          0          seq
    ......................................acauucaacgcugucgguga...................................................   4          0          seq
    ......................................acauucaacgcugucggugagu.................................................   14         0          seq
    .......................................cauucaacgcugucggugag..................................................   1          0          seq
    ...........................................................................accaccgaccguugacugu...............   3          0          seq
    ...........................................................................accaccgaccguugacugua..............   3          0          seq
    ...........................................................................accaccgaccguugacuguac.............   40         0          seq
    ...........................................................................accaccgaccguugacuguacc............   67         0          seq
    ...........................................................................accaccgaccguugacuguaccu...........   7          0          seq
```
